# Synthesis, Structural Characterization and In Vitro Immunosuppressive Activity of Quinoa Bran Soluble Dietary Fiber–Gallium Complex

**DOI:** 10.3390/foods15081415

**Published:** 2026-04-17

**Authors:** Hongyang Shu, Yichen Ai, Huajie Yin, Qiyuan Zhang, Sangguan You, Ruijuan Yang, Yunfei Ge

**Affiliations:** 1 College of Food Science and Technology, Yunnan Agricultural University, Kunming 650201, China; hongyangshu9@gmail.com (H.S.); 15911515800@163.com (Y.A.); 18887981272@163.com (H.Y.); 17387428724@163.com (Q.Z.); 2 Department of Marine Food Science and Technology, Kangwon National University, Gangneung Campus, 7 Jukheon-gil, Gangneung-si 25457, Gangwon-do, Republic of Korea; sangguanyou@gmail.com

**Keywords:** polysaccharide–metal ions complex, soluble dietary fiber, gallium (Ga), structural, immunity

## Abstract

The biological effects of dietary fiber (DF) are often associated with its chemical structure and interactions with the immune system. In this study, soluble DF (SDF) from quinoa bran was modified via gallium ion (Ga^3+^) chelation to form SDF-Ga. Results showed that gallium chelation reduced molecular weight, homogenized the polymer, and increased chain branching, forming a compact three-dimensional network. The cytotoxicity of HCT-116 colorectal cancer cells mediated by NK cells was significantly influenced by SDF-Ga, reaching 45.32% at 100 μg/mL. Key immune factors exhibited notable upregulation. Co-culture assays indicated that SDF-Ga inhibited cancer cell proliferation and migration (*p* < 0.01). In vitro assays suggested a concentration-dependent inhibition of HCT-116 cell viability, exhibiting enhanced anticancer potential compared with unmodified SDF. In summary, our results highlight that gallium chelation is an effective strategy to improve the functional properties of dietary fibers. The dual immunomodulatory and anticancer activities of the SDF-Ga complex position it as a valuable candidate for the development of novel nutraceuticals and health-promoting food products.

## 1. Introduction

Dietary fiber (DF), an important functional component of plant-derived foods, has long been recognized for its roles in maintaining intestinal health and preventing metabolic diseases [1]. Due to the influence of its physical structure, the biological effects of DF are also different, such as swelling and dilution, and are closely associated with its chemical structure, solubility, and interactions with the host microbiota and immune system [2,3]. Soluble DF (SDF) exhibits high solubility and fermentability and can be utilized by the gut microbiota to produce short-chain fatty acids (SCFAs) [4]. SCFAs, in turn, regulate intestinal epithelial barrier integrity, activate dendritic cells and T cells, and inhibit the proinflammatory pathway [5,6,7]. Moreover, the conformation of SDF also affects the binding affinity of immune receptors, such as changes in molecular weight, functional groups, etc., thereby regulating innate and adaptive immune responses [8]. Quinoa bran (Chenopodium quinoa Willd.), the by-product produced after industrial processing, has quite a lot of bioactive components such as SDF [9]. However, native SDF from quinoa bran has a compact and stable conformation with limited functional group exposure [10], which limits its interaction with immune receptors or tumor targets and restricts its bioactivity. Thus, the high-value utilization of quinoa bran has considerable nutritional and economic significance.

Metal ion modification has emerged as an effective strategy for functionalizing polysaccharides by markedly altering their physicochemical properties and biological activities through chelation [11]. The underlying mechanism involves the coordination of metal ions with electron-donating groups, such as hydroxyl, carboxyl, and aldehyde moieties, on the polysaccharide chains, forming stable cyclic or three-dimensional complexes. This coordination can disrupt the intermolecular hydrogen bonds, enhance chain flexibility, and trigger supramolecular structural rearrangements [12]. Gallium (Ga^3+^), a metal ion with unique biological effects, mimics Fe^3+^ in chemical behavior, allowing it to competitively bind to iron-binding proteins, disrupting iron-dependent metabolic processes and inhibiting tumor cell and pathogen proliferation [13,14]. In addition, Ga^3+^ regulates the immune response by activating macrophages, enhancing natural killer (NK) cells’ function, and suppressing inflammatory cytokine expression [15]. Therefore, incorporating Ga^3+^ into quinoa bran SDF via chelation could reorganize the distribution of surface functional groups and enhance its affinity for immune receptors or tumor targets, thereby improving its immunostimulatory and anticancer activities.

Previous studies have reported the immunomodulatory and anticancer effects of certain metal-polysaccharide complexes exerted through multiple mechanisms, including enhanced free radical scavenging, inhibition of reactive oxygen species (ROS)-related signaling pathways, upregulation of anti-apoptotic proteins, and activation of immune cells [16,17]. However, the structural characteristics and biological activities of quinoa bran SDF-Ga^3+^ complexes remain to be fully elucidated. We hypothesized that Ga^3+^ chelation could modify the physicochemical properties of quinoa bran SDF, potentially enhancing its interaction with immune cells and tumor targets. Therefore, this study aims to prepare the SDF-Ga^3+^ complex, characterize its structural features, and evaluate its immunomodulatory and anticancer effects in vitro.

## 2. Materials and Methods

### 2.1. Materials and Reagents

Quinoa bran was supplied by Changchun Jiayu Grain and Oil Co., Ltd. (Changchun, China). DMSO, TFA, and NaBD4 were from Sinopharm Chemical Reagent Co., Ltd. (Shanghai, China). DMEM, RPMI-1640, and McCoy’s 5A were from Qingdao Haibo Biotechnology Co., Ltd. (Qingdao, China). The 1 mg/mL 5-FU was stored at −20 °C and was purchased from Sigma-Aldrich (St. Louis, MO, USA). DCFH-DA, Annexin V-FITC/PI, and TRIzol reagents were obtained from Beyotime (Shanghai, China), and trypsin–EDTA from Sigma-Aldrich (St. Louis, MO, USA). All reagents were analytical grade, and biosafety standards and basic and clinical pharmacology and toxicology are observed in this experiment [18].

### 2.2. Preparation of Soluble Dietary Fiber (SDF)

The white quinoa (*Chenopodium quinoa* Willd.) used in this study was purchased from Changchun Jiayu Grain and Oil Co., Ltd. The grains were manually screened to remove impurities, washed three times with distilled water to remove surface dust, and then dried in a convective oven at 40 °C for 24 h. The dried quinoa bran was then obtained by separation from the grain and crushed using a crusher to obtain powder. First, the quinoa bran was degreased by taking 10 g for standby, wrapping it in tin foil, and refluxing it with 100 mL of petroleum ether at 60 °C for 4 h. The defatted quinoa bran powder was fully dissolved in distilled water and phosphate buffer. Subsequently, starch components were removed by the addition of high-temperature α-amylase. In the sample, neutral protease was added to hydrolyze proteins, and starch glucosidase was added to further decompose the residual oligosaccharide or polysaccharide structures. After enzymatic hydrolysis, the mixture was centrifuged to eliminate insoluble residues, and the clarified supernatant was subsequently collected for further use. Crude quinoa bran soluble dietary fiber (SDF) was obtained through ethanol precipitation and subsequent drying [19].

To further purify the sample, protein removal was performed by the Sevag method. Crude SDF (1.0 g) was weighed and 200 mL of distilled water were added to fully dissolve it, to which 40 mL of Sevag reagent was then added for further processing. To eliminate proteins and other impurities, it was stirred at AT for 10 min (10,000× *g*), and centrifuged to collect the aqueous phase for further purification. This procedure was repeated 10 times. Subsequently, the pH was adjusted to 8.5 (using a solution containing 5% volume fraction and 50 mL of 30% hydrogen peroxide). The sample was then subjected to a molecular weight cutoff of 3500 Da using tap water for 2 days and DW for 1 day. Afterwards, the sample was precipitated with ethanol, and the quinoa bran SDF was finally obtained by freeze-drying [18].

### 2.3. Preparation of SDF-Ga Chelate

To identify the optimal chelation efficiency, a series of preliminary trials was conducted. Based on the results, 0.2 g of gallium (III) chloride was weighed and dissolved in 100 mL of deionized water (DW) in a triangular flask. The pH was adjusted to 2.0–3.0 using a 0.3 mol/L hydrochloric acid solution to maintain the stability of ionic gallium. Then, the prepared GaCl3 solution (2 mg/mL) and SDF solution (5 mg/mL) were mixed in a 1:1 (*w*/*w*) ratio. The mixture was incubated on a shaking incubator at 50 °C for 48 h to ensure sufficient coordination between gallium ions and the functional groups of SDF. Subsequently, the pH of the mixture was adjusted to 3.0 to facilitate the precipitation and purification of the complex. The SDF-Ga complex was purified using a 3000 Da dialysis membrane (Spectrum Medical Industries, Los Angeles, CA, USA) against DW for 48 h, followed by freeze-drying to obtain the final product [18].

### 2.4. Structural Characterization of SDF and SDF-Ga

#### 2.4.1. Scanning Electron Microscopy (SEM) Measurement

The surface morphology of SDF and SDF-Ga was characterized using a Zeiss Sigma 360 SEM (Zeiss AG, Oberkochen, Germany) at an accelerating voltage of 5.0 kV and a working distance of 8.5 mm. Before observation, samples were mounted on metal stubs using conductive adhesive tape and coated with a 20 nm thick gold layer using an ion sputter (Ar gas, 15–25 mA, 90–180 s). To ensure the statistical representativeness of the results and to provide a basis for quantitative comparison, at least five random fields of view were captured for each sample at magnifications of 500× and 2000×. The micrographs were used to quantitatively assess the differences in particle fragmentation and surface architecture between the samples.

#### 2.4.2. Molecular Weight Analysis

Molecular weight parameters, including number-average molecular weight (Mn), weight-average molecular weight (Mw), and the polydispersity index (PDI, Mw/Mn), were determined via GPC (Agilent 1260 series). The separation was performed on an Agilent column (7.5 × 300 mm, 8 μm, Agilent) at a flow rate of 1.0 mL/min and a column temperature of 30 °C, using 0.1% (*w*/*w*) sodium sulfite as the mobile phase. Calibration was performed using Shodex P-82 polysaccharide standards to relate retention volume to molecular weight. Quantitative integration of the elution peaks was processed using Agilent OpenLab software (version C.01.07, Agilent Technologies, Santa Clara, CA, USA) to derive precise values for Mw and Mn. This quantitative approach allowed for a precise determination of the shift from bimodal to unimodal distribution upon Ga-complexation.

#### 2.4.3. Monosaccharide Analysis

The measurement was performed using a GC-MS system (model 6890N/MSD5973, Agilent Technologies, Santa Clara, CA, USA). A 5mg weight sample was reacted with 4M TFA and allowed to react at 100°C for 6 h, then evaporated to dryness under nitrogen and co-evaporated with methanol three times to remove borate. The residue was reduced using a freshly prepared NaBD_4_ aqueous solution. After cooling, the reaction was quenched with glacial acetic acid and co-evaporated with methanol several times to further remove borate. Mixed standards of Glc, Gal, Man, Ara, Xyl, Rha, and Fuc were used as internal standards with inositol. Quantification was determined from the normalized peak area of the internal standard and expressed in mole percent.

#### 2.4.4. Determination of Glycosidic Linkages

The glycosidic linkages of SDF and SDF-Ga were analyzed following the Hakomori method with slight modifications. Briefly, dried samples (1–5 mg) were dissolved in anhydrous DMSO under a nitrogen atmosphere, followed by methylation using powdered NaOH and methyl iodide. Extraction was conducted with methylene chloride, washed repeatedly with DW. After using 4M TFA and continuing to react at 100 °C for 4 h, NaBD_4_ and acetic anhydride were successively added. The resulting partially methylated alditol acetates (PMAAs) were dissolved in methylene chloride and subjected to GC–MS for analysis.

### 2.5. Assay of Immunostimulatory Activities

#### 2.5.1. Cell Line and Cell Culture

Both NK cells and HCT-116 cell lines were from the RIKEN Bioresource Center of the Tsukuba Institute of Science, Japan. NK cells were kept in α-Minimum Essential Medium (α-MEM, Gibco, Norristown, PA, USA), while HCT-116 cells were kept in McCoy’s 5A medium (Gibco). Subculturing was performed at an appropriate cell density (approximately 80% confluence), and all experiments were conducted under conditions where the cells were in good health, exhibited complete morphology, and were free from mycoplasma contamination [20].

#### 2.5.2. Cell Proliferation Analysis

The cell inoculation density was set as 1 × 10^6^ cells/mL (96-well plate), NK cells and HCT-116 cells were treated with SDF and SDF-Ga at different concentrations (25 to 400 μg/mL) to co-culture at 37 °C, 5% CO_2_, in a wet incubator. After 24 h, cell proliferation was assessed using the EZ-Cytox assay (or CCK-8/WST-1 assay). A total of 10 μL of EZ-Cytox reagent was added to each well and incubated for an additional 2 h. The absorbance was measured at 450 nm using a microplate reader. Determine the absorbance with sodium nitrate as the standard curve. The cell viability was calculated by comparing the absorbance values of each experimental group and the control group, which was set as 100% [21].

#### 2.5.3. Co-Culture Cytotoxicity

The uncontaminated NK cells were inoculated into the culture dish, and the samples of different concentrations were added in this experiment. After 24 h of cultivation, the NK cells were collected and co-cultured with HCT-116 cells in 96-well plates at an effector-to-target (E:T) ratio of 10:1. After incubation for 4 h, 10 µL of WST-1 reagent was added to each well, and the plate was incubated for an additional 4 h under the same conditions. The optical density (OD value) was measured using the same microplate reader as described in Section 2.5.2. The degree of cytotoxicity was calculated using the following formula:

Cytotoxicity (%) = 100(1 − (As − Ae/Am))

where As is the absorbance of the co-culture sample (NK + Target cells), Ae is the absorbance of NK cells alone, and Am is the absorbance of target cells (HCT-116) alone. These results provide the experimental basis for clarifying the mechanism of SDF and SDF-Ga in regulating NK cell-mediated cytotoxicity.

#### 2.5.4. Real-Time PCR Analysis

In this experiment, the concentration of the sample was set as SDF and SDF-Ga (25–100 μg/mL), and then inoculated on the pre-prepared inoculation plate containing NK cells (1 × 10^5^ cells/mL) for 24 h. The co-cultured samples were added with Trizol reagent (Invitrogen, Carlsbad, CA, USA) to obtain total RNA and determine its concentration. After adjusting to a uniform concentration, RNA was reverse-transcribed into cDNA. PCR (qRT-PCR) was determined on a CFX Connect Real-Time PCR System (Bio-Rad, Hercules, CA, USA) according to transcription rules [21]. The specific primer sequences used in this study are listed in Table 1.

#### 2.5.5. Inhibition of Cytotoxicity Assay Using PRR-Specific Inhibitors

To investigate the potential receptors involved, NK cells were pre-treated with anti-TLR4/MD2, anti-TLR2/MD2, and anti-CR3 monoclonal antibodies (20 μg/mL) for 2 h. Subsequently, the cells were treated with samples for another 24 h, and the cytotoxicity was measured as described in Section 2.5.3 [22].

### 2.6. Determination of Anticancer Activity

#### 2.6.1. Cancer Cell Proliferation

HCT-116 cells were incubated, and the logarithmic phase cells were 5 × 103 cells/well. SDF or SDF-Ga were diluted to 300–600 μg/mL with complete medium. The combination of cells and medium was used as the control, and the combination of medium and EZ-Cytox was used as the blank. Incubate for 24 h in a CO_2_ incubator (37 °C, 5% CO_2_). After that, add 10 μL EZ-Cytox reagent and continue to incubate for 2–4 h. The absorbance was measured at 450 nm using a microplate reader (EL-800; BioTek Instruments, Winooski, VT, USA).

#### 2.6.2. Cell Apoptosis Analysis

Annexin V-FITC/PI flow cytometry was used to quantify early apoptosis (Annexin V^+^/PI^−^), late apoptosis (Annexin V^+^/PI^−^), and necrosis (Annexin V^+^/PI^−^). Briefly, HCT-116 cells were treated with SDF or SDF-Ga (400 μg/mL) for 24 h. For the ROS-related apoptosis inhibition assay, cells were pre-treated with N-acetylcysteine (NAC, 20 mM) for 30 min before sample incubation. Subsequently, cells were harvested, washed, and resuspended in Annexin V-binding buffer containing 1.25 μL Annexin V-FITC and 10 μL propidium iodide (PI). After incubation for 15 min at room temperature in the dark, apoptotic populations were analyzed using a flow cytometer (Beckman Coulter, Brea, CA, USA) [23].

#### 2.6.3. ROS Generation Analysis

Intracellular ROS levels were determined using the DCFH-DA fluorescent probe. HCT-116 cells (2 × 10^5^ cells/mL) were seeded into 24-well plates. Cells were treated with 400 μg/mL of SDF or SDF-Ga, while 5-fluorouracil (10 μg/mL) served as the positive control. Following treatment, cells were incubated with 10 μM DCFH-DA (Beyotime, Shanghai, China) in serum-free medium for 20 min at 37 °C in the dark. After removing excess probe by washing with PBS three times, the fluorescence intensity was measured using a multi-mode microplate reader (Tecan, Männedorf, Switzerland) at an excitation wavelength of 488 nm and an emission wavelength of 525 nm [24].

### 2.7. Statistical Analysis

All experimental data were obtained from at least three independent replicates (*n* = 3) and expressed as mean ± SD. Data analysis was performed using SPSS 19.0 software. The normality and homogeneity of variance were confirmed using Shapiro–Wilk and Levene’s tests. Comparisons between groups were analyzed by one-way ANOVA with Tukey’s post hoc test. Statistical significance was set at * *p* < 0.05, while ** *p* < 0.01 and *** *p* < 0.001 indicated highly significant differences.

## 3. Results

### 3.1. Structural Characterization of SDF and SDF-Ga Complex

#### 3.1.1. Molecular Weight Distribution

**Figure 1:**
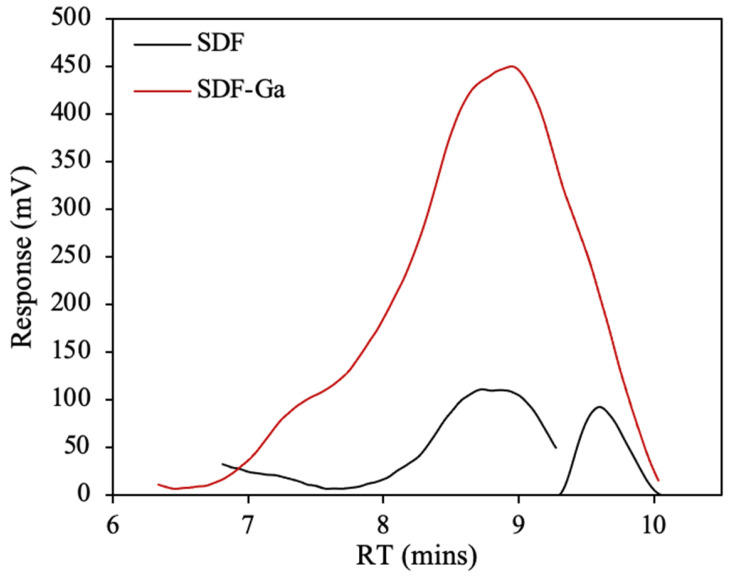
Chromatographic peak profiles of SDF and SDF-Ga.

Table 2 and Figure 1 demonstrate the significant differences in molecular weight distributions of SDF and its gallium complex (SDF-Ga). Before chelation, SDF showed a bimodal distribution with an average molecular weight (Mw) of 183.3 ± 3.8 kDa and a polydispersity index (PDI) of 6.51. Following coordination with gallium, the molecular weight of SDF-Ga quantitatively decreased to 121.6 ± 4.1 kDa, and the distribution shifted toward a more uniform unimodal profile with an increased PDI of 8.34. The chromatographic curve (Figure 1) also shows that the peak of SDF-Ga moves forward, becomes more concentrated, and shows enhanced peak response, indicating that the molecular structure and chain conformation of SDF have undergone profound changes in the process of complexation with Ga^3+^. The molecular weight of Enteromorpha prolifera polysaccharide and *Flammulina velutipes* polysaccharide decreased by 8.43% and 0.92%, respectively, indicating that the outer chain of the complex was easy to dissociate during dialysis or purification [25]. In addition, the infrared spectrum showed that the stretching vibration of hydroxyl(-OH) shifted and weakened, indicating that hydroxyl participated in the coordination reaction, resulting in the overall decline of molecular weight [26].

#### 3.1.2. Microstructure Characteristics of SDF and SDF-Ga Samples

**Figure 2:**
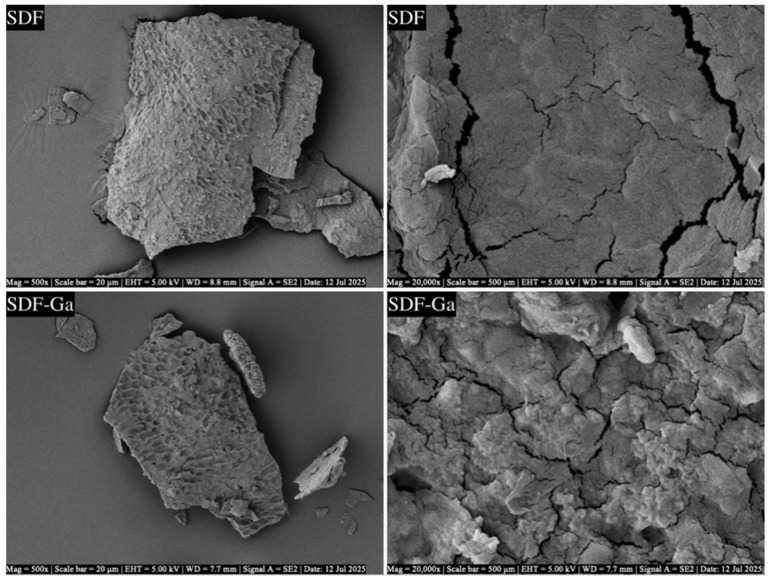
Morphological analysis of SDF and SDF-Ga. (Low- and high-magnification SEM images of SDF (**top**) and SDF-Ga (**bottom**). Images were captured at 500× and 20,000× magnifications with scale bars of 20 μm and 500 nm, respectively).

To elucidate the morphological alterations in soluble DF induced by chelation, a microstructural analysis was conducted (Figure 2). The SEM images illustrate the morphological differences between SDF and SDF–Ga at the same magnification. At low magnification (500×; scale bar = 20 μm), SDF exhibited large, flat sheet-like structures with a rough surface, prominent cracks, and honeycomb-like pores, indicating a high porosity and low compactness. In contrast, SDF–Ga displayed more compact sheet-like structures with a smoother surface, fewer cracks, partially filled pores, and significantly improved structural integrity. At a high magnification (20,000×; scale bar = 500 nm), SDF appeared as disordered and fragmented sheets with loose textures and abundant pores, whereas SDF–Ga showed a dense and uniform granular structure with tightly packed particles, presenting a more compact morphology. Quantitative assessment across five random fields of view confirmed that this structural transformation was uniform throughout the sample, characterized by a substantial reduction in particle size and the formation of uneven aggregates. These findings suggest that Ga^3+^ coordination disrupts the original ordered and porous structure of SDF, resulting in a denser and more homogeneous microstructure, consistent with previous findings that metal ion chelation promotes the structural densification and particle uniformity of polysaccharides [27].

#### 3.1.3. Determination of Monosaccharide Composition

Determination of monosaccharide composition is necessary to elucidate the structural characteristics of polysaccharides and their relationship to functional modifications. Table 3 shows significant differences in the monosaccharide composition of SDF and its SDF-Ga. Glucose and galactose are the main monosaccharides in the two samples, and they accounted for more than 60% of the total sugar content. In SDF-Ga, glucose content increased from 36.51 ± 0.75% to 38.93 ± 1.02% (*p* < 0.05), and galactose also increased slightly but statistically. Glucose and galactose residues, typically located in the main or branched chains of the polysaccharide backbone, contain abundant hydroxyl and epoxy groups that readily coordinate with metal ions, leading to their relative enrichment following chelation [28].

The mannose content significantly increased following chelation (from 7.31 ± 1.12% to 8.11 ± 0.11%, *p* < 0.05), potentially attributed to its unique spatial conformation and exposed terminal hydroxyl groups that favor coordination with gallium ions [29]. In contrast, arabinose content significantly decreased (from 13.27 ± 0.01% to 11.41 ± 0.18%, *p* < 0.05), possibly due to partial dissociation or rearrangement during the coordination process. Other minor monosaccharides, such as rhamnose, xylose, and fucose, showed no significant changes. Moreover, metal chelation may induce polysaccharide chain folding and localized degradation, altering its solubility and microenvironment. This exposes certain residues and facilitates their participation in coordination, modifying the overall monosaccharide composition [30].

#### 3.1.4. Glycosidic Linkage Analysis

Methylation analysis of SDF-Ga (Table 4) revealed a structurally diverse and highly branched polysaccharide backbone composed predominantly of glucopyranosyl (Glcp) and galactopyranosyl (Galp) residues. Notably, terminal (→1)-linked Glcp residues showed the highest relative abundance (16.08%), followed by (→4)-linked Glcp (13.88%) and (→6)-linked Glcp (8.03%), suggesting that the glucan backbone is primarily composed of →4)- and →6)-linked Glcp residues with abundant non-reducing termini. This pattern indicates a typical β-D-glucan-like structure with significant branching. Galactose residues were also prevalent, including (→1)-, (→6)-, (→4)-, (→4,6)-, and (→3,6)-linked Galp, contributing a cumulative peak ratio of approximately 33.1%. These diverse linkages imply that Galp plays a dual role in backbone extension and side chain branching. Mannopyranosyl (Manp) residues accounted for ~8.80%, mainly in →1)- and →6)-linked forms, suggesting their involvement in peripheral side chains. Arabinofuranosyl (Araf) linkages (→1)-, →3)-, and →3,4)- accounted for 10.4% of the total, indicating their localization at branching regions or terminal residues. Additionally, the →2,4)-linked rhamnose (4.14%) and → →2,3,4)-linked xylose (5.58%) showed that a small amount of other sugar residues contributed to structural heterogeneity. Collectively, the methylation patterns indicate that SDF-Ga is composed of highly branched polysaccharides with a Glcp-rich backbone and Galp- and Araf-rich side chains. The prevalence of multi-substituted Galp linkages such as →4,6)- and →3,6)-Galp further indicates a dense network of side chains, which may provide abundant coordination sites for Ga^3+^ binding. This conformation is likely to enhance the structural stability, solubility, and biological activity of the gallium–polysaccharide complexes, which further confirms that SDF and its gallium complex (SDF-Ga) had significant differences in monosaccharide composition.

### 3.2. Immunostimulatory Activities

#### 3.2.1. The Effects of SDF or SDF-Ga on NK Cell and HCT116 Cell Proliferation

**Figure 3:**
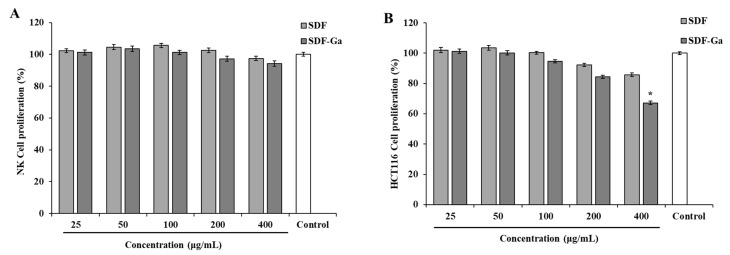
The effects of SDF or SDF-Ga on NK cell and HCT116 cell proliferation. (**A**) NK cell proliferation was detected after exposure to different concentrations of SDF or SDF-Ga (25, 50, 100, 200, and 400 μg/mL). (**B**) HCT116 cell proliferation was detected after exposure to different concentrations of SDF or SDF-Ga (25, 50, 100, 200, and 400 μg/mL). Data are expressed as the mean ± SD (*n* = 3). The asterisk indicates statistical changes (* *p* < 0.05, versus control).

Next, we studied the cytotoxicity of SDF-Ga on tumor and NK cells. In Figure 3, the proliferation of NK cells (Figure 3A) and HCT-116 cells (Figure 3B) is shown to be different at different concentrations of SDF/SDF-Ga (25, 50, 100, 200, and 400 μg/mL). For NK cells, SDF and SDF-Ga treatments maintained cell viability at approximately 95% or higher across all tested concentrations; this result is not statistically significant. Conversely, HCT-116 cells exhibited a concentration-dependent inhibition, particularly in the SDF-Ga group. At 400 μg/mL, SDF-Ga diminished the proliferation of HCT-116 cells to approximately 70% (* *p* < 0.05), whereas, at the same concentration, SDF reduced proliferation to approximately 85%. These findings indicate that SDF-Ga has a stronger inhibitory effect on HCT-116 cells than SDF does, while exerting minimal effects on NK cells.

SDF-Ga significantly inhibited the proliferation of HCT-116 colorectal cancer cells, and inhibition increased with the increase in concentration, with minimal impact on NK immune cells, even at high concentrations. This tumor-selective activity is likely associated with the intrinsic antitumor characteristics of Ga^3+^ ions. Previous studies have shown that Ga exerts its antitumor activity by disrupting iron metabolism, inhibiting DNA synthesis, and inducing apoptosis in tumor cells. As a polysaccharide carrier, SDF may enhance the uptake and stability of Ga in tumor cells, potentiating its anti-tumor efficacy. The negligible cytotoxicity of SDF-Ga toward NK cells suggests good biocompatibility and safety. Therefore, in order to determine whether SDF-Ga enhances NK cell activity and promotes apoptosis of HCT-116 cancer cells, subsequent experiments were carried out with 25 to 100 μg/mL concentrations.

#### 3.2.2. The Effects of SDF or SDF-Ga on NK Cell Cytotoxicity

**Figure 4:**
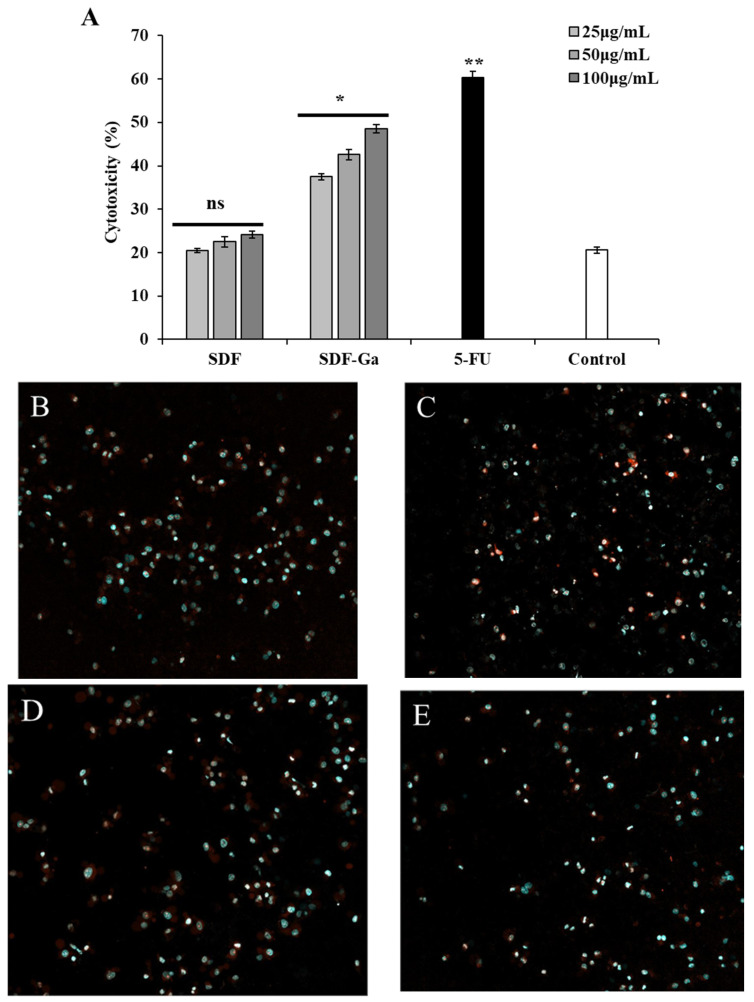
The effects of SDF or SDF-Ga on NK cell cytotoxicity. (**A**) Percentage of NK cell cytotoxicity against HCT116 cells after treatment with SDF, SDF-Ga, or 5-FU. The NK cell cytotoxicity was detected after exposure to different concentrations of SDF, SDF-Ga (25, 50, and 100 μg/mL), or 5-FU (10 μg/mL). Quantitative analysis of SDF or SDF-Ga (100 μg/mL) treated NK cells against HCT116 cells: (**B**) NK cell as a control, (**C**) SDF, (**D**) SDF-Ga, and (**E**) 5-FU. Data are expressed as the mean ± SD (*n* = 3). The asterisks indicate statistical changes (ns: not significant, * *p* < 0.05, ** *p* < 0.01, versus control).

Due to their selective cytotoxicity toward tumor cells, NK cells are regarded as promising effectors in immunotherapy. Their primary mechanism underlying immune function is cytotoxicity against cancer cells. Immune cells acquire tumoricidal activity when activated by cytokines or other stimuli [30]. Therefore, herein, we utilized two detection methods to monitor the SDF-Ga-mediated activation of NK cells to achieve tumoricidal activity. As shown in Figure 4A, gallium-complexed DF exhibited a significantly stronger ability to activate NK cells than unmodified DF. The cytotoxicity of NK cells not activated by SDF or SDF-Ga against target cancer cells was 22.15% (MD group). Significant cytotoxicity was observed in the 100 μg/mL SDF-Ga pre-treatment group relative to the control, but not in comparison with the MD group. The positive control group, 5-FU, exhibited a cytotoxicity rate of 59.05%, which demonstrated a difference from the MD group that was incredibly significant. To further verify that the complex formed between DF and gallium ions could stimulate immune cells (NK) more effectively than DF alone, thereby enhancing their cytotoxicity against target cancer cells, CQ1 was employed to monitor apoptosis in target cancer cells.

Compared with untreated NK cells, the number of apoptotic cell colonies (PI-positive) in HCT-116 cells treated with SDF-Ga for 4 h was increased, the amount of apoptosis in the SDF-treated group and the untreated NK cell group did not differ significantly. The quantity of DAPI-positive viable cells in the positive control group (5-FU) was significantly lower than that of untreated NK cells, indicating that SDF-Ga can significantly activate NK cells and result in a greater degree of apoptosis in target cells compared with SDF treatment.

#### 3.2.3. The Effects of SDF or SDF-Ga on mRNA Expression of Inflammatory Factors

**Figure 5:**
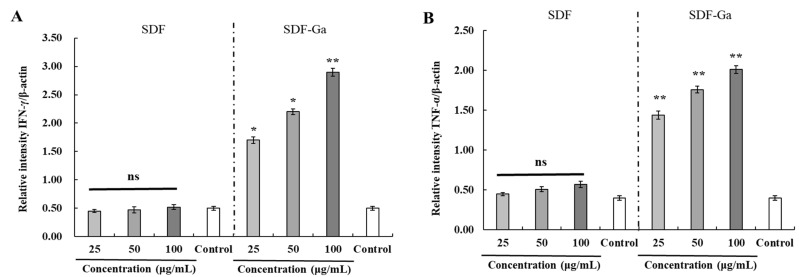

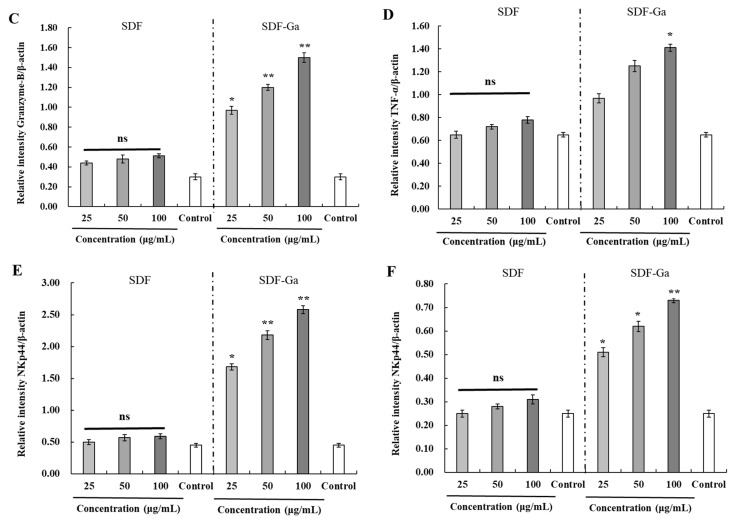
The effects of SDF or SDF-Ga on mRNA expression of inflammatory factors. The NK cell mRNA levels were detected after exposure to different concentrations of SDF or SDF-Ga (25, 50, and 100 μg/mL) and co-culture with HCT116 cells. Real-time PCR was used to detect the mRNA levels of (**A**) IFN-γ, (**B**) TNF-α, (**C**) granzyme-B, (**D**) perforin, (**E**) NKp30, and (**F**) NKp44. β-actin was used as the normalization. Data are expressed as the mean ± SD (*n* = 3). The asterisks indicate statistical changes (ns: not significant, * *p* < 0.05, ** *p* < 0.01, versus control).

Targeted tumor cells are affected by strong cytotoxicity after NK cells are stimulated and activated. Therefore, NK cell activation is crucial for effective cancer immunotherapy and the prevention of viral infections. When NK cells are activated, cytokines (IFN-γ and TNF-α), enzymes (granzyme-B and perforin), receptors and ligands (NKp30 and NKp44) are secreted [31]. Accordingly, we co-cultured NK cells with HCT-116 cancer cells using untreated NK cells as the control group. NK cells were stimulated by incubation with either SDF or SDF-Ga, followed by co-culturing. In Figure 5, SDF did not significantly induce IFN-γ and TNF-α expression, while SDF-Ga significantly upregulated their mRNA levels compared with the control group. These cytokines can bind to membrane receptors present on tumor cells to activate apoptotic signaling pathways, inducing apoptosis in the targeted cancer cells [32]. Similar to cytotoxic T cells, NK cell-mediated killing mainly depends on the serine protease granzyme family and pyrogen perforin [33]. In the SDF group, the mRNA expression levels of all indicators showed no significant upregulation, indicating weak activation ability and a lack of effective induction. Granzyme-B and perforin expression levels in NK cells, on the other hand, were markedly elevated by the SDF-Ga complex in a concentration-dependent manner. NKp30, acting synergistically with NKp46 and/or NKp44, induces an NK cell-mediated killing effect on most target cells, and is the main activating receptor for killing some tumor cells [34]. The mRNA expression levels of receptors and ligands (NKp30 and NKp44) were consistent with the results for cytokines and enzymes, indicating that the chemical modification of SDF with gallium ions enhanced their ability to stimulate NK cell activation and improved their tumor-targeting cytotoxic potential.

#### 3.2.4. The Effects of SDF-Ga on Specific Antibodies Against PRR on NK Cell Cytotoxicity

Immune cells exert cytotoxic immune responses by specifically recognizing pathogens, including viruses, bacteria, and other microbial agents, through the engagement of pattern recognition receptors (PRRs) [35]. NK cells express a diverse repertoire of PRRs, potentially allowing them to sense pathogens directly. In innate immune defense, the immune response and cytotoxic activity of NK cells are affected by Toll-like receptors (TLRs). TLRs sense exogenous pathogenic signals and activate downstream signaling pathways, thereby exerting a stimulatory effect [36,37]. Complement receptor 3 (CR3) is a transmembrane protein that acts as a major receptor, facilitating the recognition and phagocytosis of microbial pathogens. Neutrophils, NK cells, and monocytes exhibited a significant expression effect [38]. Therefore, the biological structure and function of NK cells are associated with tumor-related immune response.

**Figure 6:**
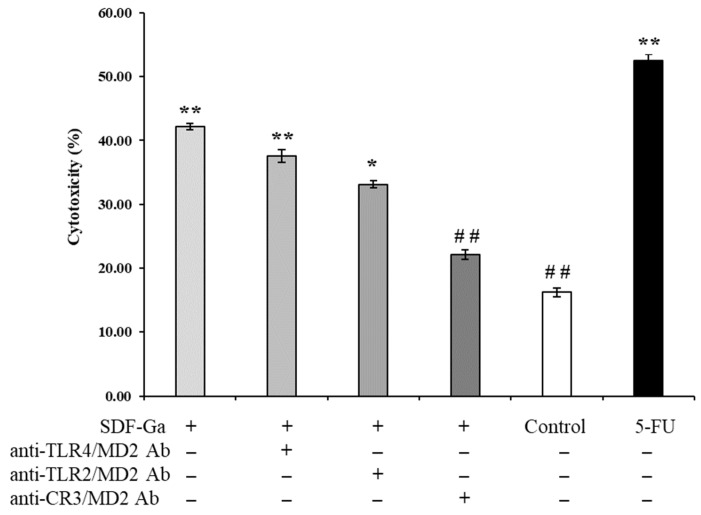
The effects of SDF-Ga on specific antibodies against PRR on NK cell cytotoxicity. The NK cell cytotoxicity was detected after exposure to the SDF-Ga (100 μg/mL) or antibodies (10 μg/mL). Data are expressed as the mean ± SD (*n* = 3). The asterisks indicate statistical changes (* *p* < 0.05, ** *p* < 0.01, versus control; and ## *p* < 0.01, versus SDF-Ga).

In this study, anti-TLR2, anti-TLR4, and anti-CR3 antibodies were used to study the potential signaling pathway of SDF-Ga-mediated NK cell activation. NK cells were pre-incubated with anti-TLR2, anti-TLR4, and anti-CR3 antibodies for 1h, and then co-cultured with HCT-116 cells after stimulation with SDF-Ga. The effect of antibody pre-treatment on NK cell killing activity was observed. In Figure 6, SDF-Ga treatment significantly increased the cytotoxic activity of NK cells against HCT-116 colorectal cancer cells. These findings suggest that SDF-Ga can promote NK cell activation and enhance their immune-mediated antitumor effects. However, pre-treatment with an anti-TLR2 antibody markedly reduced the cytotoxic capacity of SDF-Ga-activated NK cells against tumor cells, possibly due to the role of TLR2 in enhancing cytotoxic activity across various cancer cell lines (Lu et al., 2011) [39]. Additionally, pre-treatment with an anti-CR3 antibody significantly decreased the cytotoxicity of SDF-Ga-treated NK cells. In contrast, no similar inhibitory effect was observed with anti-TLR4 antibody treatment, indicating that SDF-Ga primarily activates NK cells and mediates its anti-tumor immune effects through a CR3-dependent signaling pathway.

### 3.3. Anticancer Activity

#### 3.3.1. The Effect of SDF or SDF-Ga on HCT116 Cell Proliferation

**Figure 7:**
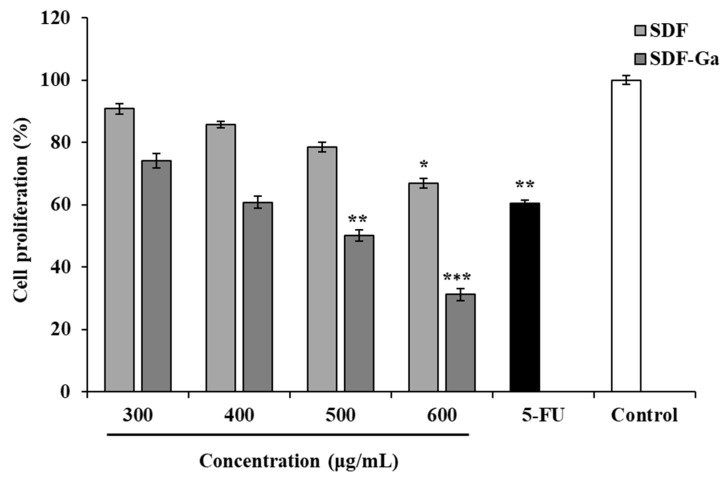
The effect of SDF or SDF-Ga on HCT116 cell proliferation. The HCT116 cell proliferation was detected after exposure to the SDF, SDF-Ga (400 μg/mL), or 5-FU (10 μg/mL). Data are expressed as the mean ± SD (*n* = 3). The asterisks indicate statistical changes (* *p* < 0.05, ** *p* < 0.01, and *** *p* < 0.001, versus control).

Our preliminary investigation showed that at low concentrations (25–100 μg/mL), quinoa bran SDF complexed with gallium ions (SDF-Ga) could activate NK cells and enhance their cytotoxicity against targeted HCT-116 cancer cells, indicating that SDF-Ga possesses certain immuno-antitumor activity under low-concentration conditions. To further evaluate the potential biological application value of SDF and SDF-Ga in suppressing tumor cell growth, the inhibitory effects of different concentrations of SDF and SDF-Ga on the proliferation of HCT-116 colorectal cancer cells were detected by the EZ-CytOx new cell viability assay kit. In Figure 7, SDF showed a relatively weak inhibitory effect on HCT-116 cells at all concentrations. When the concentration of SDF increased from 300 to 500 μg/mL, No statistically significant difference was observed relative to the control group. At a concentration of 600 μg/mL, SDF exhibited a 33.15% inhibitory effect on the proliferation of HCT-116 cells. In contrast, SDF-Ga demonstrated a concentration-dependent suppression of HCT-116 cell growth. As the concentration increased from 300 μg/mL to 600 μg/mL, the cell viability gradually decreased from 74.14% to 31.14%, and the difference was statistically significant compared with the control group. As a reference, the positive control 5-FU showed a cell viability of 60.47%. These results indicate that gallium ion chelation significantly enhanced the antitumor activity of quinoa bran SDF, improving its biological efficacy and potential for further biomedical applications.

#### 3.3.2. The Effect of SDF or SDF-Ga on ROS Generation

**Figure 8:**
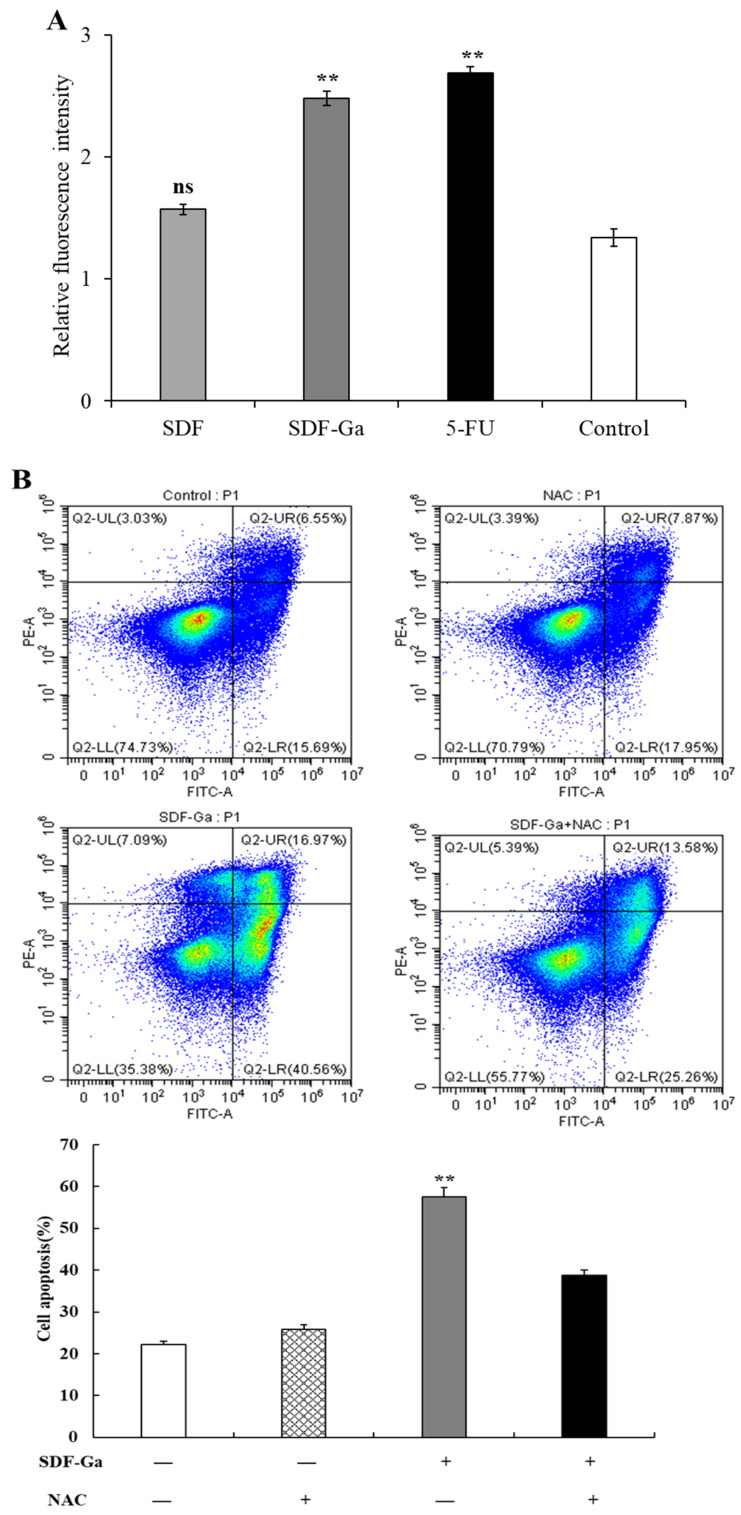
The effects of SDF or SDF-Ga on ROS generation. (**A**) The cancer cells’ ROS levels were detected after exposure to the SDF, SDF-Ga (400 μg/mL), or 5-FU (10 μg/mL). (**B**) HCT116 cells were cultured with SDF-Ga (400 μL/mL) or NAC (20 μL/mL) for 24 h, and cell apoptosis was detected by flow cytometry analysis. Data are expressed as the mean ± SD (*n* = 3). The asterisks indicate statistical changes (** *p* < 0.01, versus control) and “ns” indicates no significant difference (*p* > 0.05).

Apoptosis is an effective mechanism for inhibiting the proliferation of cancer cells; excessive ROS production has been shown to disrupt the intracellular antioxidant defense system, inducing apoptosis [39,40]. In addition, compared with normal cells, cancer cells are more sensitive to ROS levels. Evidence from clinical studies has demonstrated that many anticancer agents promote tumor cell apoptosis by elevating ROS levels beyond the cytotoxic threshold [41]. To determine whether SDF and SDF-Ga can induce HCT-116 cell apoptosis through ROS, 400 μg/mL SDF or SDF-Ga was used for 24 h, and ROS accumulation within cells was determined with an Infinite M200 Pro microplate reader. The results showed that SDF-Ga significantly induced ROS production in HCT-116 cells compared to SDF alone (Figure 8A). To further confirm whether SDF-Ga-induced apoptosis is mediated by the production of intracellular ROS, HCT-116 cells underwent a 1 h pre-treatment with NAC (ROS scavenger) followed by co-incubation with SDF-Ga. In Figure 8B, NAC treatment reduced the number of apoptotic responses induced by SDF-Ga alone, indicating that ROS generation plays a critical role in SDF-Ga-induced apoptosis of HCT-116 cells. Therefore, elevating the ROS levels in cancer cells to cytotoxic thresholds is an effective strategy for selectively targeting and killing tumor cells.

Despite the significant findings regarding the synthesis and immunosuppressive potential of the SDF-Ga complex, some limitations of this study should be acknowledged. First, the structural characterization primarily focused on macroscopic changes and specific functional groups; further high-resolution analysis, such as X-ray absorption fine structure (XAFS), could provide more precise details on the coordination geometry of Ga^3+^. Second, the evaluation of immunosuppressive activity was limited to in vitro cell models (RAW 264.7 macrophages). While these results provide valuable preliminary evidence, they may not fully reflect the complex physiological environment and metabolic transformations occurring in vivo. Future research involving animal models is necessary to validate the systemic efficacy, bioavailability, and long-term safety of SDF-Ga as a potential functional food supplement or therapeutic agent [42].

## 4. Conclusions

The structural features, immunoregulatory functions, and anticancer activities of quinoa bran-derived SDF and its gallium complex (SDF-Ga) were systematically investigated. The results indicated that gallium ions could form stable coordination complexes with SDF, which appeared to alter their molecular structures. A marked decrease and more uniform distribution in the Mw of SDF-Ga was observed. Monosaccharide composition analysis revealed increased proportions of Glc, Gal, and Man residues after chelation, while those of Fuc and Ara decreased, suggesting that Ga3+ may preferentially bind to hydroxyl-rich and conformationally stable sugar residues. Further methylation-GC-MS analysis showed an increase in the content of backbone and branched structures, such as (→4)-Glcp-(1→), (→6)-Glcp-(1→), and (→4,6)-Galp-(1→), and (→4,6)-Galp-(1→), suggesting potential for enhanced chain branching and three-dimensional network formation in SDF-Ga. In terms of biological functions, SDF-Ga exhibited no obvious cytotoxicity toward NK cells within the 25–100 μg/mL range, and inhibited HCT-116 colorectal cancer cell proliferation to a certain extent, with inhibition reaching 30–35% at 400 μg/mL (*p* < 0.05). Additionally, SDF-Ga was found to upregulate the expression of IFN-γ, TNF-α, granzyme-B, perforin, and NKp44, and other key immune factors under the experimental conditions. The co-culture of SDF-Ga and HCT-116 cells further suggested that SDF-Ga might inhibit the proliferation and migration of tumor cells, potentially mediated by ROS accumulation. A significant apoptotic response (44.71%) was observed, which was associated with the elevated expression of Bax and Caspase-3 and the suppressed expression of Bcl-2. The results also indicated that SDF-Ga activated NK cells via PRRs, particularly CR3. Collectively, Ga3+ chelation not only modified the structure of SDF but also appeared to enhance its immunomodulatory and anticancer activities, highlighting its potential applicability as a functional ingredient in food systems or as an adjuvant in cancer therapy.

## Figures and Tables

**Table 1:** The details of primers used in the real-time PCR analysis.

Gene		Primer Sequence
*IFN-ɣ*	Forward	GATGCTCTTCGACCTCGAAACAGCAT
	Reverse	ATGAAATATACAAGTTATAATCTTGGCTTT
*TNF-α*	Forward	CCCCCACAGTCAAAGACACT
	Reverse	TACAGGCTTGTCACTCGAATT
*Granzyme-B*	Forward	AGATCGAAAGTGCGAATCTGA
	Reverse	TTCGTCCATAGGAGACAATGC
*Perforin*	Forward	AGTCCTCCACCTCGTTGTCCGTGA
	Reverse	AAAGTCAGCTCCACTGAAGCTGTG
*NKp30*	Forward	GACTTCACCAGTTTAAGTAAATC
	Reverse	CTGGGAGATGAGTGAATTTCATA
*NKp44*	Forward	TCCAAGGCTCAGGTACTTCAA
	Reverse	GAT-TGTGAATCGAGAGGTCCA
*β-actin*	Forward	ATGTGCAAAAAGCTGGCTTTG
	Reverse	ATTTGTGGTGGATGATGGAGG

**Table 2:** Molecular characteristics of SDF and SDF-Ga.

Sample	Mw (kDa)		Mn (kDa)		Polydispersity Index (Mw/Mn)	
	Peak I	Peak II	Peak I	Peak II	Peak I	Peak II
SDF	183.3 ± 3.8 ^a^	4.2 ± 2.5 ^a^	28.2 ± 0.4 ^a^	3.8 ± 1.8 ^a^	6.51 ± 0.02 ^a^	1.11 ± 0.01 ^a^
SDF-Ga	121.6 ± 4.1 ^b^	/	14.6 ± 2.3 ^b^	/	8.34 ± 0.05 ^a^	/

Note: Mean ± standard deviations of triplicate analysis are given. Different superscript letters in the same column represent a significant difference (*p* < 0.05).

**Table 3:** Monosaccharide content (%) of SDF and SDF-Ga fractions extracted from Quinoa bran.

Monosaccharide	SDF (%)	SDF-Ga (%)
Rhamnose	5.64 ± 0.31 ^c^	3.95 ± 0.05 ^d^
Fucose	0.66 ± 1.58 ^a^	0.79 ± 1.03 ^c^
Arabinose	13.27 ± 0.01 ^d^	11.41 ± 0.18 ^a^
Xylose	5.31 ± 0.02 ^c^	4.75 ± 0.03 ^c^
Mannose	7.31 ± 1.12 ^b^	8.11 ± 0.11 ^a^
Glucose	36.51 ± 0.75 ^b^	38.93 ± 1.02 ^a^
Galactose	31.30 ± 0.09 ^c^	32.06 ± 0.05 ^d^

^a–d^ designate a statistical difference (*p* < 0.05) among the soluble dietary fiber extracted from Quinoa bran.

**Table 4:** Methylation analysis of SDF-Ga.

Retention Time (Min)	Methylation	Glycosidic Linkage	Peak Ratio
6.470	1,5-Di-O-acetyl-2,3,4,6-tetra-O-methyl-D-galactitol	→1)-Galp-(1→	12.40 ± 0.94
7.067	1,3,5-Tri-O-acetyl-2,4-di-O-methyl-D-arabinitol	→1)-Arap-(1→	4.854 ± 2.12
8.016	1,2,5-Tri-O-acetyl-3,4-di-O-methyl-D-arabinitol	→3)-Arap-(1→	2.071 ± 0.23
8.576	1,2,3,4,5-Penta-O-acetyl-6-O-methyl-D-mannitol	→1)-Manp-(1→	3.750 ± 0.28
8.687	1,2,5-Tri-O-acetyl-3,4-di-O-methyl-D-arabinitol	→3,4)-Arap-(1→	3.456 ± 0.64
9.346	1,2,3,4,5-Penta-O-acetyl-6-O-methyl-D-glucitol	→1)-Glcp-(1→	16.080 ± 0.14
9.717	1,2,3,4,5-Penta-O-acetyl-6-O-methyl-D-galactitol	→6)-Galp-(1→	7.979 ± 0.85
10.246	1,3,5-Tri-O-acetyl-2,4-di-O-methyl-L-rhamnitol	→2,4)-Rhap-(1→	4.144 ± 2.05
11.050	1,2,3,4,5-Penta-O-acetyl-6-O-methyl-D-glucitol	→6)-Glcp-(1→	8.034 ± 1.11
11.182	1,2,3,5,6-Penta-O-acetyl-4-O-methyl-D-glucitol	→4)-Glcp-(1→	13.877 ± 0.93
11.329	1,2,3,4,5-Penta-O-acetyl-6-O-methyl-D-mannitol	→6)-Manp-(1→	5.054 ± 1.04
11.517	1,2,4,5-Tetra-O-acetyl-3,6-di-O-methyl-D-galactitol	→3,6)-Galp-(1→	4.222 ± 0.34
12.061	1,2,3,5,6-Penta-O-acetyl-4-O-methyl-D-galactitol	→4)-Galp-(1→	3.316 ± 0.77
13.911	1,2,3,5-Tetra-O-acetyl-4,6-di-O-methyl-D-galactitol	→4,6)-Galp-(1→	5.186 ± 0.34
16.382	1,5-Di-O-acetyl-2,3,4-tri-O-methyl-D-xylitol	→2,3,4)-Xylp-(1→	5.578 ± 0.48

## Data Availability

The data that support the findings of this study are available from the corresponding author upon reasonable request.
